# In Self-Defence

**Published:** 1890-01-25

**Authors:** 


### The Doctor's Pulpit.

### IN SELF-DEFENCE.

Among the lower animals, one of the most common of all endowments is some provision for self-defence. Most persons who have visited the seaside are familiar with the creatures known as "jelly fishes" (*Medusidæ*). Another name for these animals is "sea nettles"; an appellation derived from the property which some of them possess of severely stinging any exposed part of the human body they may come in contact with. This power is due to the presence of large numbers of what are known as "thread cells," and is found on all ordinary occasions to be amply sufficient for self-defence. The beautiful corals with which we are all so familiar are outside skeletons of little animals belonging to the order *Actinozoa*, and they preserve their little colonies in perfect security from the devouring jaws of deep-sea monsters. The scaly shells of the varieties of crustaceans, such as crabs and lobsters, make a secure fortress for their bold and greedy tenants. The cuttle fishes, on the other hand, have, with one or two exceptions, an internal skeleton; and even that of a very rudimentary kind. But that these creatures can give a good account of themselves either in fight or in flight their enemies very well know. Their huge globular eyes can see round them on all sides; their tentacles, provided with suckers, are able to hold on with fatal tenacity. If they elect to fly instead of to fight they can force themselves rapidly backwards through the water, still facing the enemy; and if he proves to be too persistent and urgent in his attentions, they can eject the contents of their inkbags into the surrounding water and thus render themselves promptly invisible and safe. Most people are acquainted with the structures and methods of defence employed by land animals of the vertebrate orders. The hare trusts to her speed and to her skill in "doubling," and if she once reach the covert, is generally safe from further pursuit. The horned animals, on the other hand, keep to the open; for if they did not, their horns would speedily become entangled in the lower branches of the trees, and they would thus become an easy prey. When hard pressed in flight, instinct teaches them that if life is to be saved the enemy must be faced, and accordingly they turn round and face him, fighting hard to the bitter end.

All these circumstances have been pointed out again and again by naturalists; and it has come to be generally understood that the animal which has no means of defence is decidedly in a bad way. If it may ever be said with truth of any human creature that he is "all mankind's epitome," it may certainly be said of the human race, as a whole, that it is the epitome of the whole animal creation—that and more. Provisions for self-defence are as necessary for the man as for any of the lower animals, only they must be of a much more varied character. We do not find human beings very highly by nature endowed with physical means of defence. It is highly probable that if man had had no more intellect than the ape he would have become extinct by this time; for it is certain that with the weapons Nature has provided for him he could have made no stand even against the larger apes. It is clear, then, that since man has survived he has successfully defended himself by means other than those of flight or hand-to-hand conflict.

It is no part of our purpose to discuss from the point of view of general science the question of human methods of defence and means of survival. Almost every person who has arrived at adult age finds himself, as a matter of fact, in the midst of hostile surroundings. The human race, and particularly that part of it which speaks the English language, consists of innumerable individual personalities and wills. To the present writer it is a constant miracle that the possessors of those personalities and wills have not torn each other to pieces, or in some other way fatally extirpated each other, ages ago. When it is considered that each man's hunger must be satisfied, that other overmastering passions demand gratification, that every man's intellect, and consequently his will, differs more or less from every other man's, that each man has interests distinct and often opposed to those of all other men, and that each man is endowed with a consciousness that his own interests are to him of more importance than those of any other man, or, indeed, than those of all men together, it is obvious that a miracle is wrought when the different races and individuals of mankind are preserved from mutual extinction.

The instinct of self-preservation is probably the strongest of all human endowments. We find, however, that that instinct alone is not sufficient for self-preservation among human beings, however well it may ordinarily serve that purpose among the lower animals. For what is it that man as man is bound to preserve? Is it merely his life? It is not only his life, but his health; not only his health, but his self-respect; not only his self-respect, but his conscience and a reasonable amount of freedom for the exercise of his individual judgment and will. That this is no holiday task has been proved again and again by the self-abandonment of whole races of men to abject slavery. It is constantly demonstrated by the cringing and unmanly deference the servile tradesman pays to his rich customer; by the slavish and immoral obedience which the employed so frequently yield to the employer; by the sacrifice of judgment and conscience that the politician will make to his party or to his constituents; and by the miserable self-extinction of the voter in the presence of a wealthy patron of an opposite political creed. The men and women who make a successful defence of their complete manhood in the noblest sense are probably the few and not the many. Yet, if the greatest thinkers and profoundest seers of human story are right, it would have been better for those who have failed to make a successful defence if they had never been born.

What is the primest of all essentials for human self-defence of this complete kind? Health, undoubtedly! Health—in its largest sense of physical robustness, intellectual capacity, and moral wholesomeness. What is the man more than the brute if he have not a capable intellect; and what is the intellect more than superior cunning if it be not under the dominance of a noble morality? These are not rhetorical questions, but practical. Who thinks of Nero to-day except as of an infamous and worthless fool? And, on the other hand, who, even of the bitterest enemies of the Romish Church, fails to recognise that the fisherman Peter was of the nature of a moral antiseptic, whose virtue is by no means exhausted even at the present day? Animal health may be health of body and no more; human health, to be human and not merely animal, must include health of mind and soul. Very few people take into consideration the peculiar evils of massing human beings together in huge cities and vast communities. Most of us, however, are able to understand that bad drainage, impure air, a smoky atmosphere, and other concomitants of life in large towns impair the physical energy and make bodily life less wholesome, buoyant, and sweet. It is practically certain that this physical result of large town life is more or less commensurate with the mental and moral results. There is an impairment of the intellectual powers and of the moral energies of man, resulting from his separation from natural objects and simple ways of life, which correspond generally with the diminution of his physical buoyancy and robustness. This is scientifically accurate; and as important as it is true.

All town men who spend a month or two in the country every year know well the difference between the buoyant and cheerful energy of the country-holiday time and the frequent langour, depression, and irresolution of the nine or ten months spent in the smoky and impure atmosphere of the large town. Unhappily, no illustration lies ready to hand which will as clearly show the debilitating effect of town life upon the mental and moral parts of man. Yet that the average of mental energy is greatly lowered may be readily understood from the exceedingly small number of independent intellects of sufficient energy to impress themselves on their contemporaries, compared with the much larger proportion of such intellects that were found in the small populations of the Greek cities in the five or six centuries immediately preceding the Christian era. The truth is, whether men will believe it or not, that town life deadens the body, deadens the mind, and deadens the soul. Those who have mental powers equal to the occasion clearly see and strongly desire some means of defence against this too urgent pressure. The most effectual means is, of course, "flight;" the method of the hare when hard pressed by the superior speed and threatening teeth of the greyhound. But for the majority flight is not possible. We must stand at bay, with our backs to some friendly wall, and meet our various enemies face to face. Standing thus we become vividly conscious of the immeasurable advantage of stored resources, of physical energy, mental readiness and capacity, and clear moral purpose—in short, of that all round health which is the natural and necessary complement of our all round character and position in the scale of being. Charged and energised with this threefold health we can fight steadily, strongly, calmly, and enduringly. The battle in these times is usually both long and strong, and victory frequently delays her approach. Thousands go down and disappear for want of the power of physical endurance. Quite as many thousands fail for lack of steadily improving brain capacity. But, perhaps, most of all are smitten, crushed, and routed because of their poverty of clear and steadfast moral purpose. The real strength of man, we repeat, is his health—his threefold health; and without this no start, however good, no advantages on the way, however great, no stroke of luck, however wonderful, can save him from defeat. Like all other creatures, his means of defence must be commensurate with every possible occasion, or final victory can never crown his brow.

